# The Role of HOX Transcription Factors in Cancer Predisposition and Progression

**DOI:** 10.3390/cancers11040528

**Published:** 2019-04-12

**Authors:** Bo Li, Qilai Huang, Gong-Hong Wei

**Affiliations:** 1 Shandong Provincial Key Laboratory of Animal Cell and Developmental Biology, School of Life Sciences, Shandong University, Qingdao 266237, China; libo1051@163.com; 2 Faculty of Biochemistry and Molecular Medicine, Biocenter Oulu, University of Oulu, 90220 Oulu, Finland

**Keywords:** HOX, cancer susceptibility, risk SNP, coding mutation, regulatory SNP, mechanism

## Abstract

Homeobox (HOX) transcription factors, encoded by a subset of homeodomain superfamily genes, play pivotal roles in many aspects of cellular physiology, embryonic development, and tissue homeostasis. Findings over the past decade have revealed that mutations in HOX genes can lead to increased cancer predisposition, and HOX genes might mediate the effect of many other cancer susceptibility factors by recognizing or executing altered genetic information. Remarkably, several lines of evidence highlight the interplays between HOX transcription factors and cancer risk loci discovered by genome-wide association studies, thereby gaining molecular and biological insight into cancer etiology. In addition, deregulated HOX gene expression impacts various aspects of cancer progression, including tumor angiogenesis, cell autophagy, proliferation, apoptosis, tumor cell migration, and metabolism. In this review, we will discuss the fundamental roles of HOX genes in cancer susceptibility and progression, highlighting multiple molecular mechanisms of HOX involved gene misregulation, as well as their potential implications in clinical practice.

## 1. Introduction

The homeobox genes encode a highly conserved family of transcription factors that play essential roles in embryonic development and tissue homeostasis. In humans, there are four *HOX* gene clusters, *HOXA*, *HOXB*, *HOXC*, and *HOXD*, located on different chromosomes, at 7p15, 17q21.2, 12q13, and 2q31 loci, respectively. Each cluster consists of 9 to 11 *HOX* genes arranged in order. A total of 39 transcription factors are encoded by *HOX* genes and regulate a series of downstream target genes in a precise manner. The spatial and temporal expression pattern of these HOX transcription factors and their controlled genes is the main mechanism defining the organogenesis of limbs and organs along the anterior-posterior (A-P) axis during embryonic development of flies and vertebrates [1,2]. Accumulating evidence shows that functional abnormalities of HOX transcription factors play critical roles in the development and progression of many types of cancers [1,2].

Both mutation and aberrant expression can alter the function of the HOX transcription factor by gene regulation, and subsequently affect downstream events of cancer development. Mutations in the HOX DNA binding domain and cofactor-interaction domain may alter the structure and function of protein, thereby leading to an aberrant capability of DNA binding [3] and protein–protein interaction [4], respectively. In cancer, the deregulated *HOX* gene expression has been widely recognized as a driving force in tumorigenesis [1]. Either up-regulation or down-regulation of *HOX* genes have both been reported to be associated with cancer under various conditions, where *HOX* genes act as tumor suppressors or proto-oncogenes depending on cancer type. For example, *HOXA9* was found to be overexpressed in leukemia, but downregulated in breast cancer. As a proto-oncogene, high expression levels of *HOXA9* are often associated with increased cancer risk [2]. Aberrant expression of HOXA9 was proven to play critical roles in the development of acute leukemia through reprogramming the epigenome or synergizing with other transcription factors and signaling pathways, thereby considered as one of the driving forces in leukemogenesis [5,6]. On the other hand, as a tumor suppressor, HOXA9 inhibits the tumor phenotype by regulating the expression of *BRCA1* in breast cancer [7]. In clinical settings, lower expression of *HOXA9* is greatly associated with elevated tumor invasion, metastasis, and patient mortality [7]. In addition to this, the deregulation of many *HOX* genes has been found in a variety of cancers, and often reported in association with an increased cancer risk and poor survival rate of cancer patients [1,2].

*HOX* genes also play increasingly important roles in genetic predisposition for many types of cancers. A previous large-scale twin study provided solid evidence that hereditary factors made a significant contribution to colorectal, breast, and prostate cancer. In addition, the research also revealed suggestive evidence of limited heritability with leukemia and cancers of the stomach, lung, pancreas, ovary, and bladder [8]. With a wide application of exome sequencing and genome-wide association study (GWAS), substantial independent susceptibility loci marked with single nucleotide polymorphism (SNP) have been discovered for nearly all types of cancers, such as colorectal [9], breast [10], prostate [11], lung [12], cervical cancer [13], acute lymphoblastic leukemia [14], chronic lymphocytic leukemia [15], and so forth. A combination of these risk mutations may define individuals with different inherited susceptibility to developing cancers [16]. These risk genetic alterations also lay the groundwork for personalized precision cancer medicine. Among the genes, somatic or germline mutations in *HOX* genes have proven to influence cancer susceptibility. Further, HOX transcription factors often recognize and execute genetic information buried in cancer risk loci. In this review, we will systematically introduce the progress in deciphering the roles of *HOX* genes in cancer susceptibility and progression as well as the underlying mechanisms. We also briefly discuss clinical implications of HOX proteins as cancer therapeutic targets.

## 2. HOX Transcription Factors in Cancer Predisposition

### 2.1. Coding Genetic Mutations in HOX Genes 

**Figure 1:**
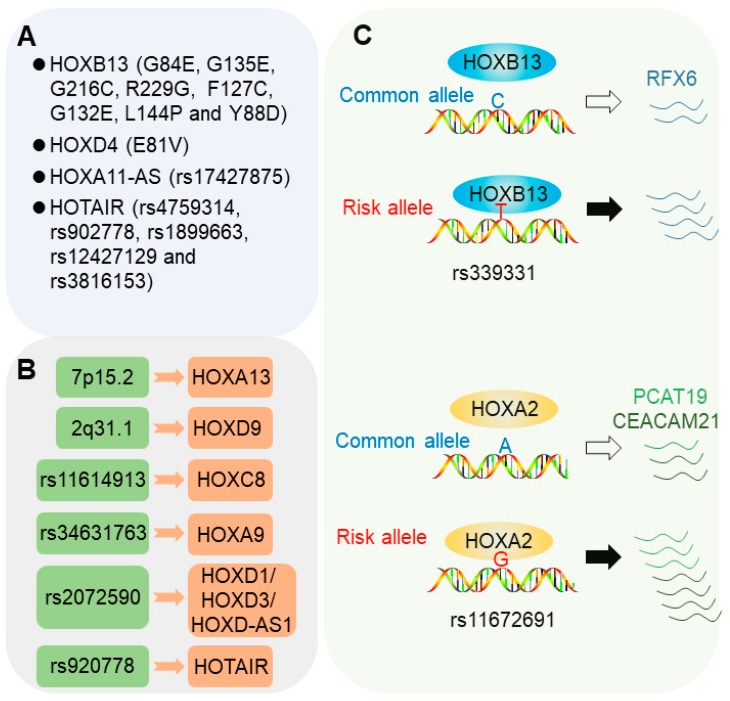
Involvement of *HOX* genes in cancer susceptibility. (**A**) Coding mutations in HOX transcription factors and HOX locus long non-coding RNAs (lncRNA) conferring increased cancer risk. (**B**) Risk loci conferring increased cancer predisposition through *HOX* deregulation. Several known cancer risk loci harboring transcriptional regulatory regions can subsequently regulate the expression of *HOX* genes. (**C**) HOX transcription factors deciphering altered regulatory genetic information of risk single nucleotide polymorphism (SNP). The HOX transcription factors, HOXB13 and HOXA2, bind the risk allele with higher affinity compared to the protective allele, thereby leading to the upregulated expression of *RFX6* and *PCAT19*/*CEACAM21*, respectively, and conferring prostate cancer susceptibility.

Coding mutations in *HOX* genes have been widely observed in association with cancer predisposition. Current evidence indicates that this type of mutation mostly locate in the HOX transcription factors, *HOXB13* and *HOXD4*, as well as a long non-coding RNAs (lncRNA), *HOXA11-AS*, from the antisense strand in the homeobox A cluster (Figure 1A). 

#### 2.1.1. HOXB13 Mutations

*HOXB13* is a homeobox B transcription factor gene and is known to be important in prostate development and tumorigenesis. Multiple studies reported a significant association of *HOXB13* with many types of cancers [17,18,19,20,21]. Remarkably, the inherited mutations in *HOXB13* have been widely observed for a genetic contribution to prostate cancer risk. Specifically, several missense rare mutations in *HOXB13*, including G84E, Y88D, L144P, G216C, and R229G, have been identified in association with increased prostate cancer susceptibility through targeted germline DNA sequencing of the 17q21-22 region in a cohort of 94 prostate cancer patients [22]. In particular, the rare, but recurrent *HOXB13 G84E* mutation is strongly associated with an increased risk of familial prostate cancer in European descents and subsequently found to be highly associated with prostate cancer risk in additional populations [23,24,25,26,27,28,29,30]. Interestingly, the other *HOXB13* mutation pattern differs among populations. Two variants, R229G and G216C, of *HOXB13* were found in African descent [22]. The *HOXB13* G135E mutation was discovered in association with increased prostate cancer risk of Chinese men [31]. Two additional *HOXB13* F127C and G132E mutations were identified among Japanese men with prostate cancer [32]. The other two mutations, L144P and Y88D, were observed in the prostate cancer cell line, LNCaP and LAPC4, respectively [22].

Intriguingly, several studies report that the *HOXB13* G84E mutation is not only associated with increased risk of prostate cancer, but also significantly associated with an elevated risk of leukemia, and cancers of the bladder, breast, and kidney [25,28,33,34,35], indicating that this prostate cancer susceptibility gene, *HOXB13*, may also play key roles in the predisposition to a variety of cancers. 

Even though the underlying mechanisms of these mutations in promoting carcinogenesis are still unknown, their potential impact could be inferred based on the domain function in protein–DNA and protein–protein interactions. The *HOXB13* gene has two highly conserved MEIS (myeloid ecotropic viral integration site) binding domains on exon 1, and one homeodomain located in exon 2 [36]. A functional *HOXB13*–MEIS1 interaction plays pivotal roles in modulating cellular proliferation and gene expression in prostate cancer [37]. Both G84E and Y88D mutations are located in the first MEIS interacting domain, while L144P and G135E mutations reside in the second MEIS interacting domain [22,31]. Thus, these mutations in MEIS interacting domains may affect the HOXB13 function through an alteration of its binding ability to MEIS cofactors or subsequent target DNA sequence recognition. Consequently, this will affect the expression of downstream target genes [31]. However, the most recent study shows that the G84E mutation does not influence the capability of the first MEIS interacting domain in HOXB13 to physically interact with MEIS1 in a pull-down assay [37], suggesting that G84E is not a loss-of-function mutation and might influence HOXB13 DNA binding specificity and cofactor interacting profiles in a subtle manner. The other mutations, R229G and G216C, are located in the N-terminal portion of the homeobox domain and affect highly conserved amino acid residues [22]. Two additional variants, F127C and G132E, are near the HOXB13 N-terminal domain [32]. Computational modeling analysis of the HOXB13 transcription factor indicates that these coding mutations might introduce structural changes in the protein. For example, the mutants, G84E and G135E, may lead to promoted protein stability and an increased half-life, thereby conferring increased cancer susceptibility [38]. Despite efforts devoted to elucidating the impact of *HOXB13* mutations, investigation on clinical prostate tumor tissues shows that *HOXB13* gene expression at both mRNA and protein levels does not differ between samples carrying the variant and wild-type allele [39].

Intriguingly, the cooperation between the rare *HOXB13* mutation and other risk factors may play an important role in promoting cancer risk. Strong chromatin binding of HOXB13 at gene regulatory regions of CIP2A was observed, and the G84E mutation further promoted this chromatin binding in the immortalized benign prostate cell line, RWPE-1 [40]. RNA interference experiments further confirmed that HOXB13 functionally promotes *CIP2A* transcription. More importantly, the simultaneous presence of HOXB13 (G84E) and the common CIP2A (R229Q) variant confers higher prostate cancer risk and disease aggressiveness, as well as poor prognosis [40]. Nevertheless, detailed biological function and mechanism of these *HOXB13* gene mutations still need to be explored.

#### 2.1.2. HOXD4 Mutations

Germline missense mutation in another *HOX* transcription factor, *HOXD4*, was also detected to be associated with an increased risk of childhood acute lymphoblastic leukemia (ALL) [41]. The E81V mutation leads to a partial loss-of-function, defined by reduced transcriptional activity at the autoregulatory enhancer of the *HOXD4* gene. This mechanism might be involved in the occurrence of childhood ALL [41].

#### 2.1.3. HOX Locus lncRNAs

Except for the aforementioned protein-coding *HOX* genes, two highly conserved lncRNA, *HOXA11-AS* at the homeobox A region and *HOTAIR* at homeobox C region, have also been reported for cancer susceptibility. *HOXA11-AS* with a minor allele T of exonic variant, rs17427875, inhibits cell survival, proliferation, migration, and invasion to a greater extent than the common allele A does in epithelial ovarian cancer [42]. As revealed in a meta-analysis, three SNPs, rs4759314, rs902778, and rs1899663, in *HOTAIR* are also the genetic predisposition factors in breast cancer, cervical cancer, and ovarian cancer [43]. Two further SNPs in *HOTAIR*, rs12427129 and rs3816153, are associated with hepatocellular carcinoma susceptibility too [44]. It is worth mentioning that many lncRNAs have been frequently found to impact cancer susceptibility [45], raising the question of whether additional *HOX* locus lncRNAs are involved in cancer predisposition and progression.

Collectively, these findings highlight the difficulties in the functional investigation of cancer risk-associated *HOX* coding mutations, and raise a possibility to perturb these mutations using advanced genome-editing tools to limit predisposition to cancers in the clinical preventive settings.

### 2.2. Risk Loci Influencing HOX Gene Expression

GWASs have thus far identified hundreds of common variants associated with cancer predisposition. According to systems annotation of the GWAS catalog database, about 93% of the risk SNPs are located in the non-protein coding regions of the genome, including intronic and intergenic regions [46]. There is increasing evidence to show that these SNPs are significantly enriched in DNase I hypersensitive sites and cistromes of transcription factors, such as HOXB13 [47,48], and are likely to act as regulatory elements to alter the expression of target genes, such as the *HOX* family member, *HOXA11* [48]. Thus, these SNPs function potentially as regulatory variants.

Until recently, several regulatory SNPs were reported to influence cancer risk through regulating the expression of *HOX* genes. Misregulated expression of *HOX* genes may lead to changes of the downstream gene expression and signaling pathways that play fundamental roles in cancers. This is in line with the observations that the expression levels of *HOX* genes are often found to be overexpressed or downregulated in many types of cancers due to various genetic and epigenetic mechanisms [1,49]. Here, we focus on several cancer susceptibility loci that may impact disease risk through misregulating the expression of *HOX* genes (Figure 1B).

#### 2.2.1. 7p15.2 Locus

The 7p15.2 locus with three SNPs, rs10486567, rs67152137, and rs7808935, has been found in association with an increased susceptibility of prostate cancer [50,51,52]. Encouraged by our previous report [48], a recent functional study identified a long-range chromatin interaction between the risk region of the 7p15.2 locus and the *HOXA13* gene, located ~873 kb away [53]. Deletion of the risk region harboring several prostate cancer risk-associated SNPs using CRISPR (clustered regularly interspaced palindromic repeats)-Cas (CRISPR-associated proteins)-mediated genome editing resulted in a loss of one anchor point of the repressive chromatin loop, which may subsequently alter the three-dimensional chromatin structure and cause upregulation of *HOXA13* and *HOTTIP* in the *HOXA* locus, leading to genome-wide transcriptomic changes [53]. Together, this study demonstrated that *HOXA13* is also a target gene transforming the roles of risk regulatory SNPs at the 7p15.2 locus that influences prostate cancer susceptibility.

#### 2.2.2. 2q31.1 Locus

Another example comes from the multiple SNPs at the 2q31.1 locus that have been reported in GWAS analysis for an association with an increased risk of mucinous ovarian carcinoma (MOC) [54] and high-grade serous epithelial ovarian cancer (HGSOC) [55]. In the mechanistic studies, a chromatin loop spanned 31 to 55 kb of the genomic region with the *HOXD9* promoter and SNPs at this locus identified using chromosome conformation capture analysis (3C) [54,55]. Subsequently, the risk SNP, rs711830, genotype was markedly associated with the expression of *HOXD9* in an expression quantitative trait loci (eQTL) analysis. Ectopic expression of *HOXD9* in MOC cells resulted in a significant increase in anchorage-independent growth [54]. In *HGSOC*, another SNP, rs2857532, located at this locus was defined as a leading causal variant, which may influence chromatin binding of the HOMEZ, BEN, and RelA-p65 transcription factors and subsequent alteration of HOXD9 expression [55]. Consistent with this, HOXD9 overexpression in the immortalized ovarian surface of epithelial cells significantly increases anchorage-independent growth, shortens population-doubling time, and reduces contact inhibition [55]. These studies together suggest that the *HOXD9* gene mediates the function of risk SNPs at the 2q31.1 locus, conferring increased susceptibility and tumor cellular transformation of MOC and HGSOC.

#### 2.2.3. 2q31 Allele rs2072590

The minor allele of rs2072590 at 2q31 was discovered in association with an increased risk of ovarian cancer (OC) [56]. This SNP lies in the non-coding DNA region downstream of *HOXD3* and upstream of *HOXD1* in the *HOXD* locus [56] and tags 19 genetic variants according to HaploReg analysis [57]. Both *HOXD1* and *HOXD3* genes have been reported for their involvement in cancer development [56]. Bioinformatics analysis plus functional annotation showed that rs2072590 together with tagged SNPs may lead to OC susceptibility through regulation of the expression of *HOXD1* and *HOXD3* [56,57]. Interestingly, the SNP, rs2072590, is also mapped within a lncRNA, namely *HOXD*-*AS1*, located between *HOXD1* and *HOXD3* in the *HOXD* cluster. Accumulating evidence reveals critical roles of *HOXD-AS1* in cancer development and progression through different mechanisms [58]. Here, the 2q31 SNP, rs2072590, might play regulatory roles in fine-tuning the expression of *HOXD-AS1*, thereby contributing to OC predisposition and progression.

#### 2.2.4. rs11614913 Locus

The SNP, rs11614913, in the miR-196a2 locus was reported to be associated with risk of childhood ALL [59] and glioma [60] in a Chinese population. *HOXC8* is the potential target gene of hsa-miR-196a2 according to a comprehensive analysis using three bioinformatics methods [59]. Rs11614913 risk allele C increases the expression levels of mature mir-196a2 and may affect the binding of mature miR-196a2 to its target mRNA in childhood ALL [59]. However, in glioma, rs11614913 polymorphism does not significantly affect the expression of mature miR-196a2; rather, it takes effect through altering its target gene, *HOXC8*, expression [60]. This finding raises another layer of complexity for a risk SNP either directly or indirectly through a miRNA to influence the expression of a potential cancer susceptibility gene, *HOXC8*.

#### 2.2.5. rs34631763 Locus

The last example is relevant to the SNP, rs34631763, within growth factor independence 1(Gfi1) that functions as a DNA binding transcriptional repressor. It is known that Gfi1 represses transcription by recruiting histone-modifying enzymes, such as lysine-specific histone demethylase 1A (LSD1), G9a (EHMT2, euchromatic histone lysine methyltransferase 2), and histone deacetylases (HDACs), to target gene promoters [61,62]. The SNP, rs34631763, in the Gfi1 gene exon was considered to be associated with acute myeloid leukemia (AML) risk in humans [63]. This missense variation introduces amino acid substitution from serine (GFI1-36S) to asparagine (GFI1-36N) at position 36 of protein Gfi1 [63]. In contrast to GFI1-36S, the GFI1-36N variant lacks the ability to bind its target gene that encodes the leukemia-associated transcription factor, HOXA9, and is unable to modify histone modifications that regulate *HOXA9* expression [64,65]. Finally, the GFI1-36N variant depresses the *HOXA9* expression by altering the epigenetic histone modification, which is consistent with the observation of frequently elevated *HOXA9* expression levels in AML patients carrying the variant [64]. This study indicates a novel mechanism by which a cancer risk variant contributes to the *HOXA9* overexpression, which may lead to epigenome reprogramming and protein–protein interaction to promote leukemogenesis [5,6].

#### 2.2.6. rs920778 Locus

The SNP, rs920778, located within the intron 2 region of *HOTAIR*, was reported to have a significant association with an increased risk of esophageal squamous cell carcinoma (ESCC) in a Chinese population. It can act as an intronic enhancer element to regulate the expression of *HOTAIR*. Compared with normal allele C, the risk allele T of the SNP, rs920778, significantly increased ESCC risk by upregulating *HOTAIR* expression [66]. 

### 2.3. Risk SNPs Modulating Chromatin Binding of HOX Transcription Factors

Current evidence emerging from functional elucidation of regulatory risk SNPs show that transcription factors are usually involved in the recognition and execution of genetic information implicated in cancer risk-associated genetic variations, thereby leading to altered gene expression and increased cancer susceptibility [46,48]. Under most conditions, the risk allele influences the DNA-binding affinity of given transcription factors, resulting in altered enhancer or promoter activity and causing varied downstream gene expression, which may finally confer increased cancer susceptibility [47,48,67]. As described in the following sections, several transcription factors are altered in DNA binding by given causal risk variants conferring cancer susceptibility and progression [47,68] (Figure 1C).

#### 2.3.1. 6q22 Allele rs339331

The SNP, rs339331, at the 6q22 locus has been reported to be associated with increased prostate cancer risk in multiple populations, including Japanese, African American, and European descent as well as Chinese men [47]. Functional studies demonstrated that this variant resides in a canonical *HOXB13*-binding site defined by bioinformatic and ChIP-seq (chromatin immunoprecipitation sequencing) analysis. The prostate cancer risk-associated T allele at rs339331 increases chromatin binding of HOXB13 to an active transcriptional enhancer, conferring allele-specific upregulation of the target gene, *RFX6* [47,69] (Figure 1C). Epigenome and transcription activator-like effector nuclease (TALEN)-mediated genome editing assays further demonstrated the direct roles of rs339331 in regulating HOXB13 chromatin binding activity and the expression of *RFX6* [69]. In the clinical setting, RFX6 upregulation in human prostate cancer correlates with tumor progression, metastasis, and risk of biochemical relapse [47]. Together, this study presented the first example of a regulatory risk SNP being responsible for prostate cancer pathogenesis through cooperation with the prostate-lineage-specific transcription factor, HOXB13, to regulate a novel oncogene, *RFX6*. 

#### 2.3.2. 19q13 Allele rs11672691

In contrast to indolent prostate cancer, the aggressive form of the disease usually indicates poor prognosis. The SNP, rs11672691, at the 19q13 locus was identified in association with aggressive prostate cancer risk in a European population [70] and prostate cancer specific mortality in a large US cohort [71]. Further genetic association analysis in a Finnish cohort of prostate cancer demonstrated that the allele G of rs11672691 is markedly associated with advanced tumor stage, prostate-specific antigen (PSA) progression, and the development of castration-resistant prostate cancer, the hallmark clinical features of aggressive prostate cancer susceptibility [68]. A follow-up functional study revealed that *HOX* transcription factor, HOXA2, plays essential roles in the causal actions and biological effects of this variation. The SNP, rs11672691, resides in an active enhancer element and the risk G allele increases the chromatin binding of HOXA2, which subsequently promotes the expression of *PCAT19* and *CEACAM21* (Figure 1C), which may contribute to the aggressive phenotype of prostate cancer [68]. Interestingly, an additional study discovered a rs11672691-mediated promoter-enhancer switching mechanism driving the expression of lncRNA *PCAT19* and thus the initiation and progression of aggressive prostate cancer [72]. The transcription factors’, NKX3.1 and YY1, DNA binding are altered by the 19q13 alleles, including rs11672691 and rs887391. Thus, these results showed that HOXA2 and additional transcription factors mediate the regulatory effect of the risk SNP at the 19q13 locus on *PCAT19* and *CEACAM21* and eventually lead to aggressive prostate cancer susceptibility [73] and also raise new questions of how these transcription factors compete for the binding to the SNP region.

Together, these tumor-type-specific contributions of HOX transcription factors in cancer susceptibility may serve as potential targets for inventing new therapeutic interference in global cancer risk prevention. It is therefore evident that the deregulation of *HOX* genes across these cancer risk loci promotes cancer susceptibility, initiation, and progression to advanced stages. 

## 3. *HOX* Genes Mediate Effects of Other Genetic and Epigenetic Variation

**Figure 2:**
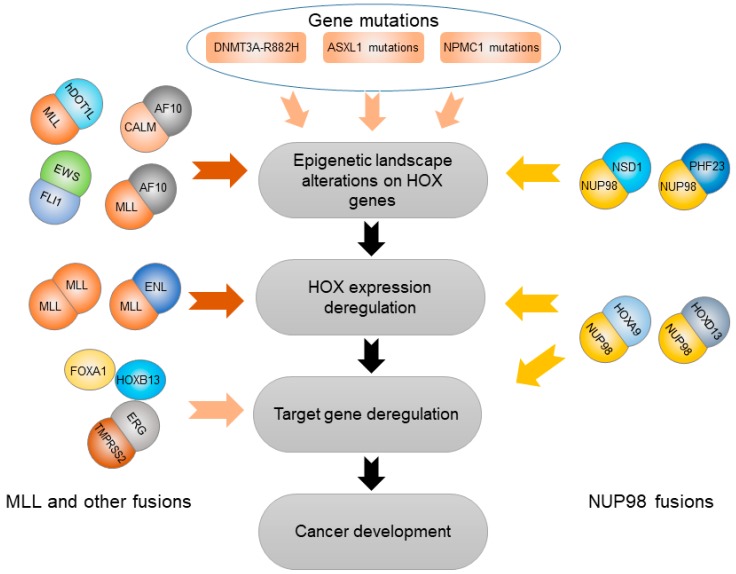
*HOX* gene-involved functional somatic variations in cancer development. Deregulation of *HOX* gene expression resulting from somatic variations plays an important role in cancer development. These somatic variations mainly include epigenetic alteration, gene mutations, and gene fusions induced by chromatin translocation.

Beside the important roles in affecting cancer susceptibility caused by germline genetic variations, the *HOX* genes also mediate the effects of a wide variety of somatic variations at both genetic and epigenetic levels. These kinds of somatic variations mainly include an abnormal epigenetic status that alters *HOX* gene expression. Here, we will discuss how somatic gene mutations in *DNMT3A*, *ASXL1*, and *NPMC1* regulate the expression of *HOX* genes through DNA methylation and histone modifications as well as gene fusions related to HOX transcription factors driving cancer progression (Figure 2).

### 3.1. Abnormal Epigenetic Alteration Affecting HOX Genes

DNA methylation is a pivotal epigenetic mechanism for defining cellular identity and regulating the activity of gene regulatory elements, including promoters and enhancers [74,75]. Aberrant DNA methylation patterns are critical in the development of all types of cancers [76]. In particular, changes in DNA methylation patterns influence *HOX* genes that commonly occur in many types of cancers [77,78,79,80,81]. Hypermethylation of the promoter-located CpG island usually leads to transcriptional inhibition and is involved in the inactivation of many tumor suppressor genes, such as *HOXA4* in chronic lymphocytic leukemia (CLL) [81]. In addition, *HOTAIR* plays an important role in epigenetically remodeling chromatin states. *HOTAIR* can recruit polycomb repressive complex 2 (PRC2) to induce *H3K27me3* modification in specific polycomb group target genes to decrease gene expression, such as *HOXD* gene clusters [82,83]. Hence, altered DNA methylation is likely to be a major potential mechanism in dysregulating *HOX* gene expression in cancer, thereby contributing to cancer development. For example, the tumor suppressor gene, *HOXA4*, was observed to be hypermethylated at promoter-associated CpG islands and correlated with low levels of *HOXA4* expression in CLL [81]. In contrast, the most recent study of genome-wide DNA methylation profiles in 30 normal tissues and 35 solid tumors found that gene-body DNA hypermethylation is greatly associated with elevated expression of *HOX* oncogenes, thus providing additional epigenetic mechanism on the regulation of *HOX* genes in cancers [49]. Further, accumulating evidence suggests that methylation alteration in *HOX* genes could serve as a prognostic marker in cancer therapy [77,78,80]. For example, promoter methylation of *HOXA9* has been associated with prognostication and can serve as potential predictive biomarker for cisplatin chemotherapy resistant bladder cancer [84].

### 3.2. Somatic Gene Mutations Deregulating HOX Transcription Factors 

#### 3.2.1. DNMT3A-R882H

Mutation R882H in DNA methyltransferase 3A (DNMT3A) was frequently found in hematological cancer, and up to 60% of patients with acute myeloid leukemia (AML) carry this mutation in heterozygous [85]. The DNMT3A-R882H variant acts in a dominant negative manner and results in a disrupted methylation function, which could subsequently upregulate both *HOXA* and *HOXB* cluster genes that are crucial for leukemogenesis [86]. Mechanically, DNMT3A-R882H directly binds to the *HOXA* gene cluster, therefore inducing DNA hypomethylation as well as H3K27 acetylation and promoting transcriptional activation of Meis1, Mn1, and HOXA that are required for DNMT3A-R882H-mediated AML progression [87]. In addition, DNMT3A-R882H significantly accelerated the progression of leukemia in the presence of other known mutations, such as NRAS-G12D, NPM1c, or IDH1R132H coexisting with the DNMT3A mutation in human AMLs, providing a susceptible genetic background in epigenetically misregulating the expression of *HOX* genes [87].

#### 3.2.2. ASXL1 Mutation

Recurrent somatic mutations in the addition of sex combs-like 1 gene (*ASXL1*) was often found in patients of myeloproliferative neoplasms and AML [88,89]. ASXL1 physically interacts with EZH2, a core member of polycomb repressive complex 2 (PRC2), leading to genome-wide histone modification of H3K27me3, including the genomic region at the posterior *HOXA* cluster. Similar to the *ASXL1* loss condition, these somatic mutations also result in the exclusion of *H3K27me3* and *EZH2* from the *HOXA* cluster [88]. Once the *H3K27me3* epigenetic signature was lost at *HOXA* clusters, *HOXA* gene expression significantly increased, in particular for *HOXA9* and *HOXA10*. Mechanistically, *ASXL1* loss-of-function mutations upregulates the expression level of *HOXA* genes by altering their methylation profile, leading to the development of cancer [88].

#### 3.2.3. NPM1 Mutation

Somatic mutations in the *NPM1* gene that encodes nucleophosmin are commonly discovered in human AMLs, accounting for about 35% of AML patients [90]. These mutations named *NPM1c* destroy the N-terminal nucleolar localization signal of nucleophosmin and produce a novel nuclear export signal, resulting in an anomalous cytoplasmic localization of the mutant nucleophosmin. Expression of the most common form of *NPM1c* in a conditional knock-in mouse model causes overexpression of several *HOXA* genes [90]. Further evidences show that in AML cells, the anomalous cytoplasmic localization of NPM1c regulates *HOXA* gene expression though histone acetylation of H3K27 at *HOX* gene super-enhancers. Either nuclear relocalization or targeted degradation of NPM1c can result in immediate downregulation of *HOX* genes and promotes differentiation of AML cells [91], suggesting an alternative therapeutic way to *HOX* genes in human AMLs carrying *NPM1* mutations.

### 3.3. Gene Fusions Cooperating with HOX Transcription Factors

#### 3.3.1. TMPRSS2-ERG (T2E) Fusion

Structural rearrangements of TMPRSS2-ERG (T2E) are present in over 50% of human prostate cancer and lead to aberrant activation of the ERG transcription factor [92,93]. In addition, the occurrence of T2E fusion is significantly associated with aggressive prostate cancer [94]. In an elegant recent study, T2E was found to physically interact with HOXB13 and FOXA1, thereby inducing T2E-specific cis-regulatory landscape in T2E-positive prostate cancer compared with non-T2E cases. Furthermore, a T2E-specific epigenomic program leads to activation of NOTCH signaling, raising a possibility of targeting T2E-positive cancers through antagonization of the NOTCH pathway [93]. These results indicate that the HOX transcription factor, HOXB13, plays important roles in mediating the oncogenic effect of T2E initiating prostate cancer development.

#### 3.3.2. *NUP98* Gene Fusion

Another example of a HOX transcription factor cooperating gene fusion is observed in nucleoporin 98kDa (*NUP98*) gene fusions that result from chromosomal translocation associated with multiple hematoplastic malignancies [95,96,97]. *NUP98* gene fusion usually encodes a fusion protein that retains the N-terminal of NUP98 with potential for transcriptional activation [96]. *NUP98* could fuse to at least 28 different genes, including multiple *HOX* family members [96]. *NUP98* fusion with HOXA9 was found to be co-localized with MLL1 on the chromatin of the *HOX* gene promoter region [97]. Furthermore, NUP98–HOXA9 (NHA9) could induce aberrant expression of dozens of genes playing roles in primary human CD34+ hematopoietic cell proliferation and differentiation [98]. Another fusion gene, NUP98–HOXD13 (NHD13) plays roles in inducing thymocyte self-renewal via Lmo2 and its critical cofactor, Lyl1 [99,100]. On the other hand, some *NUP98* fusions with genes other than *HOX* influence the development of leukemia by affecting epigenetic landscapes across the *HOX* genes, and subsequently leads to aberrant *HOX* gene expression. For example, *NUP98*–*PHF23* (*NP23*) fusion is associated with multiple hematological cancer [95]. Mechanically, *NP23* binds to a specific subset of H3K4me3-enriched chromatin sites, including at *HOXA*, *HOXB*, and *MEIS1*, and drug-targeted inhibition of H3K4me3 downregulates the expression of these target genes, leading to rapid and selective cell death of *NP23*-expressing myeloblasts [95]. Another example of non-*HOX* relevant *NUP98* fusion, *NUP98*–*NSD1* (nuclear receptor-binding SET domain protein 1), can upregulate the expression of *HOXA7*, *HOXA9*, *HOXA10*, and *MEIS1* as oncogenes in human AMLs [101]. Mechanically, NUP98–NSD1 binds directly to the regulatory genomic elements near *HOXA7* and *HOXA9*, and subsequently maintains histone acetylation and methylation of H3K36, consequently preventing transcriptional suppression of the *HOXA* cluster mediated by EZH2 during differentiation [101]. In conclusion, *HOX* genes are frequently engaged in *NUP98* fusion, thus mediating cancer progression, by acting as fusion partner genes of *NUP98*, such as *HOXA9* and *HOXD13*, or by mediating the effect of other NUP98 fusions as their target genes in a wide range of hematologic malignancies.

#### 3.3.3. MLL and Other Gene Fusions

The mixed-lineage leukemia (*MLL*) gene encodes a large histone methyltransferase possessing H3K4 methyltransferase activity, thereby actively regulating the expression of the genes, including *HOX* family members [102]. *MLL* translocation with its partner genes is highly involved in leukemogenesis through the regulation of *HOX* gene expression [102]. For example, in *MLL*-rearranged leukemia, the *MLL* oncogene promoted myeloid transformation genetically relies on *HOXA7* and *HOXA9* [103]. One of these fusions, MLL–ENL may cause leukemia by regulating the abnormal expression of *HOXA4*–*A11* in the *HOXA* cluster [104]. Another two fusions, MLL–hDOT1L and MLL–AF10 induce H3K79 hypermethylation at the *HOXA9* locus and subsequently upregulate *HOXA9* expression [105]. Mechanically, the *H3K79* hypermethylation and subsequent dysregulation of *HOXA* and *MEIS1* expression caused by the MLL–AF10 fusion oncoprotein involves recruitment of DOT1L through direct physical interaction of DOT1L–AF10 to the *HOXA* gene cluster [106]. Similarly, CALM-AF10 fusion resulting from *t* (10; 11) (p12; q23) translocation causes *H3K79* hypomethylation at the *HOXA5* locus by recruiting DOT1L [107]. In addition to histone methylation at *H3K79*, forced dimerization of *MLL* also recruits accessory transcription factors to the assembly transcriptional activation complex for activation of *HOX* gene expression [108]. Overall, *HOX* gene deregulation plays an important role in MLL/AF10 fusion-induced leukemogenesis. Therefore, it can be appreciated that while these gene fusions relevant to *HOX* transcription factors are fundamental in key stages of cancer development, their mechanistic functions can be exploited to repress tumorigenesis.

#### 3.3.4. EWS-FLI1 Fusion

EWS-FLI1 fusion is the hallmark of Ewing′s sarcoma and plays important oncogenic roles in malignant transformation. It was reported that EWS-FLI1 can reprogram the epigenome, in particular through recruitment of epigenetic regulators that facilitate chromatin opening and activate gene expression [109]. Interestingly, Ewing′s sarcoma indicates a unique *HOX* profile that includes aberrant upregulation of posterior *HOXD* genes. This aberrant elevation of *HOXD* gene expression is associated with loss of the H3K27me3 mark and gain of the H3K4me3 mark, which is mediated by EWS-FLI1 fusions. Thus, EWS-FLI1 can contribute to EWS-ETS-driven sarcoma genesis and maintenance by deregulating *HOX* gene expression in Ewing′s sarcoma, similar to MLL-fusion-driven leukemogenesis [110].

## 4. *HOX* Genes in Cancer Progression

As described above, the roles of *HOX* genes in cancer predisposition and development largely involve deregulation of the *HOX* gene as well as HOX transcription factor downstream target genes. The consequent effects of deregulated *HOX* genes in carcinogenesis can be explained as an expansion of their normal function. Based on numerous evidences about the *HOX* gene function in cancer progression, their roles can be classified into seven aspects, including angiogenesis, autophagy, differentiation, apoptosis, proliferation, invasion, and metastasis a well as metabolism that are briefly described in Table 1.

### 4.1. Angiogenesis

Angiogenesis plays key roles in the progression of solid tumors. Several *HOX* genes have been shown to function in promoting angiogenesis of solid tumors. HOXB7, HOXB9, and HOXA11 antisense RNA (HOXA11-AS) are involved in promoting angiogenesis by upregulating pro-angiogenic genes’ expression, including interleukin-8 and angiopoietin-2 [111,112,114,115,116]. HOXB7 overexpression is associated with enhanced expression of angiogenic genes in the breast cancer cell line, SKBR3, indicating that HOXB7 is a critical factor upstream of pro-angiogenic genes [111]. More evidences were observed in multiple myeloma expressing *HOXB7* to regulate myeloma pro-angiogenic properties [112]. ChIP-seq assays have uncovered hundreds of HOXB7 chromatin binding sites in the breast cancer cell line, BT-474, with ectopic expression of HOXB7 [113], thus providing a new avenue to a deep understanding of the function of HOXB7 in driving breast cancer progression and maybe multiple myeloma. HOXB9 is another potent driver of angiogenesis, promoting angiogenic recruitment by tumor cells [115]. Suppression of EGR1 and HOXB9 could result in global downregulation of genes involved in angiogenesis pathways in multiple ovarian and renal tumor models [114]. Nanoliposome-mediated delivery of microRNA-192 is indicated as an effective therapeutic for suppressing tumor angiogenesis mechanistically through downregulation of EGR1 and HOXB9 expression in tumors [114]. Another example of HOX-involved angiogenic promotion is lncRNA HOXA11 antisense RNA, named as *HOXA11*-*AS*, which was significantly overexpressed in non-small cell lung cancer (NSCLC) [116]. Tumor formation experiments revealed that *HOXA11*-*AS* promotes angiogenesis in several lung cancer cell lines [116]. In contrast with these angiogenesis-promoting *HOX* genes, the other *HOX* family members, such as *HOXA5*, were considered as antiangiogenic genes [117]. The sustained expression of HOXA5 results in downregulation of many pro-angiogenic genes and upregulation of anti-angiogenic genes in stationary endothelial cells (ECs) [118]. Mechanistically, the presence of MicroRNA-130a could reduce the anti-angiogenic activity of ECs by directly targeting the 3′-UTR of *HOXA5* [117]. Taking these observations together, in a clinical translational view, suppressing the expression of *HOXB7*, *HOXB9*, *HOXA11*-*AS*, and microRNA-130a, or maintaining the expression of *HOXA5* and microRNA-192, provides a potential therapeutic strategy to restrain tumor-associated angiogenesis and thus inhibit the growth of tumors.

### 4.2. Autophagy

Autophagy is a survival-promoting biological process that recycles aged or malfunctioning intracellular proteins and organelles and provides substrates to sustain essential metabolism in starvation and stress. Autophagy also plays important roles in the development of tumors and has been shown to have two paradoxical functions in cancer [174]. Some cancers can be inhibited by autophagy, and some rely on autophagy for survival [175]. Multiple *HOX* genes are involved in the regulation of autophagy process in cancers. For example, in human glioblastoma cells, *HOXC9* is an indicator of poor prognosis and inhibits transcription of the *DAPK1* gene through direct binding to its promoter during autophagy process [119]. Silencing of *HOXC9* could release the inhibitory effect on the *DAPK1* gene and initiate autophagy by activating *DAPK1*-Beclin1 pathway [119]. MicroRNA-193a-3p was proven to suppress cancer development by silencing multiple genes, including *HOXC9* [120]. Hence, promoting cell autophagy by directly silencing HOXC9 protein expression is a promising new cancer therapeutic strategy.

Another example of *HOX* genes in autophagy was observed in a direct inhibition of *HOXC6* with miR-185, promoting apoptosis as well as autophagy through inhibition of the TGF-β1/mTOR pathway in nasopharyngeal carcinoma [121]. Besides *HOX* transcription factors, *HOX* transcript antisense RNA (*HOTAIR*) also plays a regulatory role in autophagy and is associated with the invasion and metastasis capacities of several types of cancers. Silencing *HOTAIR* inhibits cell autophagy, proliferation, and epithelial–mesenchymal transition (EMT) through suppression of the Wnt signaling pathway and an enhancement of the sensitivity to radiotherapy [122,123].

### 4.3. Differentiation

Various *HOX* genes play pivotal roles in cell differentiation and a less differentiated stage is strongly associated with more aggressive tumor behaviors [176]. All the *HOXA* genes except *HOXA2* and *HOXA5* induce delayed hematopoietic differentiation in primary hematopoietic cells [124]. For example, HOXA9 is extensively active in blocking differentiation of hematopoietic and lymphoid cancer, and participates in the characteristic myeloid differentiation block in MN1 (Meningioma 1) leukemia [125]. Moreover, NUP98–HOXA9 fusion can confer long-term proliferation and blockaded differentiation in human primary CD34+ hematopoietic cells [98]. In addition, HOXA9 can collaborate with MEIS1 to inhibit hematopoietic cell differentiation [126]. Similarly, cooperation of HOXA9 and FOXC1 can enhance the blockade of monocytic lineage and B-lineage differentiation [127]. Moreover, *HOXA10* expression blocks tumor differentiation in prostate cancer while driving histotype differentiation and progression in ovarian endometrioid adenocarcinoma [128,129]. Another example comes from HOXB8 that incompletely blocks DMSO-induced granulocytic differentiation of HL-60 cells [130]. In comparison with these *HOX* genes, increased HOXC9 expression is associated with neuroblastoma differentiation and better prognosis in neuroblastoma patients. HOXC9 upregulation induced by retinoic acid (RA) can cause growth arrest and neuronal differentiation in neuroblastoma cells by upregulating neuronal differentiation genes and downregulating cell cycle promoting genes [131]. Intriguingly, another HOX transcription factor, HOXA5, can also be upregulated by RA and mediate retinoid differentiation therapy to block colon cancer progression. Elevated expression of HOXA5 strongly reduced tumor growth and prevents metastasis through forcing cancer stem cell differentiation [132,133]. It is worth mentioning that, in addition to *HOX* transcription factors, lncRNA HOTAIR overexpression may also affect differentiation and aggressiveness of urothelial carcinoma cells, but in a cell-type dependent manner [134].

### 4.4. Apoptosis

*HOX* genes can function both as an apoptosis-promoter and apoptosis-suppressor for cancer development. For example, accumulating evidence show that *HOXA5* and *HOXA10* are apoptosis-promoter genes. HOXA5 overexpression is associated with apoptosis in many cancers, including breast cancer [135,136], leukemia [137,138], osteosarcoma [139], lung [140], and cervical cancer [141]. In breast cancer cells, overexpression of HOXA5 promotes cell apoptosis by upregulating p53 expression [135] or activating caspase 2 and caspase 8 [136]. In addition, HOXA5 is also involved in RA-mediated apoptosis and cell growth inhibition [142]. Similarly, HOXA10 upregulation can also lead to increased p53 expression and induce apoptosis in ER positive breast cancer cell lines [143]. In contrast, *HOXA9* and *HOXC6* are considered to be apoptosis-suppressor genes. In T-cell acute lymphoblastic leukemia (T-ALL), *HOXA9* acts as an oncogene by cooperating with JAK3/STAT5 at the transcriptional level through upregulating the expression of downstream genes, such as *PIM1* and activator protein-1 (*AP*-*1*), thereby affecting cell survival and apoptosis [147]. Moreover, HOXA9 could also eliminate Meis1a-mediated apoptosis rather than Pbx1b-mediated apoptosis [126]. Notably, HOXC6 plays an important anti-apoptotic role by regulating *BCL-2* expression [144,145] in human head and neck squamous cell carcinoma and cervical cancer, and suppressing *NEP*/*MME* and *IGFBP*-*3* genes [146] in prostate cancer. Evidence that *HOX* genes could function differentially in cell apoptosis in certain cancers needs to be explored further in order to clearly demonstrate which cancer types will be suitable for HOX-targeted therapy in apoptotic signaling pathways.

### 4.5. Proliferation

Cancer cells are known as immortalized cells that could have unlimited proliferation and never die under appropriate conditions. Most of the *HOX* genes actively participate in cell proliferation in many cancers. In breast cancer, HOXA1 can stimulate cell proliferation and survival by activating the p44/42 MAPK (mitogen-activated protein kinase) signaling pathway [148] or the NF-κB pathway [149]. HOXA9 can directly drive the expression of IGF1 (insulin-like growth factor 1), which subsequently plays a key role in activating the insulin/IGF signaling pathway and other downstream signaling cascades. Functionally, proliferation and survival are preferentially affected in HOXA9-induced leukemia [150]. Besides, high expression of HOXB7 can promote cell proliferation and growth in colorectal [151] and hepatocellular carcinoma [152]. In colorectal cancer cells, HOXB7 is capable of inducing acceleration of G1-S transitions by activating PI3K/AKT and MAPK pathways, resulting in upregulation of p27Kip1 and cyclin D1 [151]. Moreover, upregulated *HOXC6* expression enhances the proliferation of gastrointestinal carcinoids cells through activation of the oncogenic AP-1 signaling pathway [153]. In addition, overexpressed oncogene *HOXB3* can lead to increased proliferation in NCI-H1437 and A549 cells [154,177]. HOXB3 could activate *DNMT3B* expression and subsequently lead to promoter methylation and repression of the gene, *RASSFA1* [154], eventually eliminating the proliferation inhibition. Similarly, HOXD3 has also been shown to increase the proliferation and anti-apoptosis activity by activating genes associated with the MAPK/AKT cell signaling pathways in hepatocellular carcinoma [155]. HOXB9 expression could be upregulated by sustained ERK5 signal activity and actively participate in proliferation and anti-apoptosis in HL cell lines [156].

On the other hand, some *HOX* genes, including *HOXC5*, act as a proliferation inhibiting gene in cancer. In thymoma and testicular germ cell tumor (TGCT), expression of HOXC5 plays key roles in suppressing the activity of hTERT [157], a protein subunit of telomerase, which is often abnormally activated and involved in proliferation in cancer [158]. Thus, HOXC5 expression could prevent tumorigenesis by inhibiting the proliferation of adult somatic cells [157].

Intriguingly, the function of the *HOXA10* gene is quite comprehensive, and acts as both a proliferation enhancer and proliferation inhibitor in cancers. HOXA10 overexpression could stimulate the proliferation of primitive myeloid progenitor cells, resulting in myeloid leukemia development [159], but also inhibit the proliferation during G2/M phases in testicular cancer cell models [160]. Collectively, these context-specific roles of HOXA10 in cancer cell proliferation serve as a cautionary reminder of therapeutic targeting of HOXA10. More evidence needs to be explored to determine which cancer types are suitable for therapeutic interference of cell proliferation with HOXA10 and other HOX proteins.

### 4.6. Invasion and Metastasis

Invasion and metastasis are the most common causes for mortality of cancer. *HOX* genes can function as invasion and metastasis-suppressor genes in cancer development. It has been revealed that multiple *HOXA* genes were involved in promoting invasion in breast cancer cells through the *HMGA2*/*TET1*/*HOXA* signaling pathway [161]. The expression of TET1 or HOXA9 significantly reduced the bone metastasis of breast cancer cells [161]. In addition, downregulation of *HOXA10* expression in endometrial carcinoma cells is responsible for their invasive behavior, which might be due to the effect of HOXA10 on the inhibition of EMT by inducing the expression of epithelial cell adhesion molecule E-cadherin [162]. HOXB1 and HOXB3 expression downregulated by microRNA-10a could facilitate invasion and metastasis in pancreatic cancer cells [163]. While overexpression of microRNA-10b accounts for invasive and metastatic behavior in metastatic breast cancer by inhibiting synthesis of HOXD10 protein at the post-transcriptional level [156,164,165,166]. Downregulation of HOXD10 in cancer results in downregulation of microRNA-7 expression and upregulation of *PAK1* expression, therefore promoting invasion and metastasis [167].

In contrast, other *HOX* genes, such as *HOXB7*, can function as invasion and metastasis-inducer genes in cancer development. HOXB7 overexpression contributes to tumorigenesis, tumor migration, and invasion through the induction of EMT in epithelial cells [168]. Also, HOXB7 overexpression induces invasive and metastatic breast cancer by activating the TGFβ signaling pathway [169]. In addition, increased metastases induced by mircoRNA-196b-5p in colorectal cancer is partially dependent on the regulation of *HOXB7* and *GALNT5* expression [170]. Besides the *HOX* transcription factors, lncRNA *HOXA11-AS* expression is positively correlated with the migration and invasion ability of gastric cancer cells [171]. Collectively, the mechanisms by which the HOX transcription factors described above regulate tumor invasion and metastasis are likely to be divergent, but the specific suppressive and inducing roles need to be further investigated as cancer-context-dependent therapeutic targets.

### 4.7. Metabolism

Metabolic pathways that support cell growth are altered and are not uniform in cancer cells [178]. Hypoxic tumor cells preferentially metabolize glucose to produce and release lactic acid through glycolysis, while other normal tumor cells use lactic acid as the substrate of mitochondrial oxidative phosphorylation (OXPHOS) [178]. It has been reported that HOXA9 [172] and HOXC8 [173] are involved in glycolysis and play important role in cancer metabolism. *HOXA9* can function as a tumor suppressor gene though downregulation of the *HIF-1α* gene in cutaneous squamous cell carcinoma (cSCC) [172]. Given the essential role of HIF-1α in glucose metabolism [179], glycolysis is inhibited by the tumor suppressor, HOXA9 [172]. The expression of microRNA-365 can downregulate HOXA9 expression by directly binding to its 3′ UTR, thus raising a possibility to therapeutically target this microRNA in cSCC with low levels of HOXA9 [172]. Moreover, ectopic expression of HOXC8 can downregulate glycolysis-related genes, upregulate TCA cycle-related genes, and subsequently inhibit nasopharyngeal carcinoma progression [173]. Thus, sustained expression of HOXA9 and HOXC8 may provide a potential therapeutic strategy to inhibit tumor growth in glycolysis-exuberant cancer.

## 5. Conclusions

In this paper, the function and mechanism of *HOX* genes in cancer predisposition and progression were discussed. Briefly, several germline coding mutations in *HOX* genes, and common genetic variations in gene regulatory elements that regulate *HOX* expression or are recognized by given *HOX* transcription factors could lead to increased cancer susceptibility. By contrast, *HOX* genes also mediate the oncogenic effect of other genetic and epigenetic variations, including an abnormal epigenetic profile on *HOX* genes, somatic mutations in other genes, and gene fusions that can regulate the expression of *HOX* genes through the establishment of abnormal epigenetic modification. The deregulated *HOX* genes might subsequently cause cancer progression from seven tumor-relevant aspects, including angiogenesis, autophagy, differentiation, apoptosis, proliferation, invasion, and metastasis as well as metabolism as briefly summarized in Table 1. Notwithstanding the challenge of deciphering the cancer-context-specific roles of these HOX transcription factors and developing anti-*HOX* therapies in cancer settings, future cancer clinical treatment plans based on these findings can likely be identified. Thus, further studies are demanded to fully illustrate the function and mechanisms by which *HOX* genes contribute to cancer predisposition and progression before these efforts can be eventually translated into the development of new strategies for precision cancer medicine.

## Figures and Tables

**Table 1:** Overview of *HOX* genes that contribute to the seven aspects of cancer development and progression.

Progression	*HOX* Gene	Tumor Cells Type	Function
Angiogenesis	*HOXB7* [111,112,113]	Breast cancer Multiple myeloma	Upregulated HOXB7 drives angiogenic gene expression
	*HOXB9* [114,115]	Ovarian cancer Renal cancer Breast cancer	Downregulated HOXB9 attenuates angiogenic gene expression
	*HOXA11*-*AS* [116]	NSCLC	Upregulated HOXA11-AS promotes angiogenesis
	*HOXA5* [117,118]	ECs	Sustained HOXA5 expression downregulates angiogenic genes and upregulates anti-angiogenic genes
Autophagy	*HOXC9* [119,120]	Glioblastoma	HOXC9 acts as a transcription inhibitor to directly binding to the promoter of *DAPK1*
	*HOXC6* [121]	NPC	Downregulated HOXC6 promotes apoptosis and autophagy by inhibiting the TGF-β1/mTOR pathway
	*HOTAIR* [122,123]	Cervical cancer; Breast cancer; Chondrosarcoma	Downregulated HOTAIR inhibits autophagy
Differentiation	*HOXA* clusters (except *HOXA2* and *HOXA5*) [124]	Hematopoietic cells	*HOXA* genes except *HOXA2* and *HOXA5* induce delayed hematopoietic differentiation
	*HOXA9* [98,125,126,127]	Hematopoietic and lymphoid cancer.	HOXA9 involves in blocking differentiation NUP98–HOXA9 fusion, cooperation of HOXA9 with either Meis1 or FOXC1 inhibit differentiation
	*HOXA10* [128,129]	Prostate cancer; OEA	HOXA10 blocks or promotes differentiation in a cancer-type-dependent manner
	*HOXB8* [130]	HL-60 cells	HOXB8 blocks DMSO-induced granulocytic differentiation
	*HOXC9* [131]	Neuroblastoma	HOXC9 promotes neuronal differentiation
	*HOXA5* [132,133]	Colon cancer	Upregulated HOXA5 promotes differentiation of cancer stem cells
	*HOTAIR* [134]	Urothelial carcinoma	HOTAIR overexpression may affect differentiation state
Apoptosis	*HOXA5* [135,136,137,138,139,140,141,142]	Breast cancer; Leukemia; Osteosarcoma; Lung and cervical cancer	HOXA5 could activate apoptosis by upregulating p53 expression or activating caspase 2 and caspase 8; HOXA5 is involved in RA-mediated apoptosis
	*HOXA10* [143]	Breast cancer	HOXA10 could activate apoptosis by upregulating p53 expression
	*HOXC6* [144,145,146]	HNSCC; Cervical cancer; Prostate cancer	HOXC6 plays an important anti-apoptotic role through regulating the expression of bcl-2 or suppressing *NEP*/*MME* and *IGFBP*-3 genes
	*HOXA9* [126,147]	Leukemia	HOXA9 functions as an apoptosis suppressor by cooperating with JAK3/STAT5; HOXA9 could eliminate Meis1a-mediated apoptosis
Proliferation	*HOXA1* [148,149]	Breast cancer	HOXA1 promotes cell proliferation and survival by activating p44/42 MAPK signaling pathway or NF-κB pathway;
	*HOXA9* [150]	Leukemia	HOXA9 upregulates Igf1 to promote proliferation and survival
	*HOXB7* [151,152]	Colorectal cancer Hepatocellular carcinoma	HOXB7 promotes cell proliferation and growth by accelerating G1-S transitions
	*HOXC6* [153]	Gastrointestinal carcinoids cells	HOXC6 promotes cell proliferation by activating the oncogenic AP-1 signaling pathway
	*HOXB3* [154]	NCI-H1437 cells A549 cells	HOXB3 promotes cell proliferation through silencing gene *RASSFA1*
	*HOXD3* [155]	Hepatocellular carcinoma	HOXD3 promotes proliferation and anti-apoptosis by activating MAPK/AKT cell signaling pathways
	*HOXB9* [156]	HL cell lines	HOXB9 upregulated by ERK5 signal promotes proliferation and anti-apoptosis
	*HOXC5* [157,158]	Thymoma; TGCT	HOXC5 inhibits proliferation by inhibiting hTERT expression
	*HOXA10* [159,160]	Myeloid leukemia; Testicular cancer	Overexpressed HOXA10 stimulates the proliferation in myeloid leukemia; HOXA10 also inhibits cell proliferation during G2/M phases in testicular cancer cells
Invasion and Metastasis	*HOXA9* [161]	Breast cancer cell	HOXA9 expression could reduce bone metastasis
	*HOXA10* [162]	Endometrial carcinoma	HOXA10 suppresses invasion by inhibiting EMT
	*HOXB1* and *HOXB3* [163]	Pancreatic cancer	HOXB1 and HOXB3 downregulation facilitates invasion and metastasis
	*HOXD10* [156,164,165,166,167]	Breast cancer	HOXD10 downregulation suppresses invasion
	*HOXB7* [168,169,170]	Breast cancer	HOXB7 overexpression induces invasive and metastatic by activating the TGFβ signaling pathway
	*HOXA11*-*AS* [171]	Gastric cancer	HOXA11-AS expression promotes metastasis and invasion
Metabolism	*HOXA9* [172]	cSCC	HOXA9 inhibits glycolysis by negatively regulating HIF-1α
	*HOXC8* [173]	Nasopharyngeal carcinoma	HOXC8 downregulates glycolysis-related genes and upregulates TCA cycle-related genes

Abbreviations: HOXA11-AS, HOXA11 antisense RNA; NSCLC, non-small cell lung cancer; ECs, endothelial cells; DAPK1,Death Associated Protein Kinase 1; NPC, nasopharyngeal carcinoma; TGF-β, transforming growth factor-β; mTOR, mammalian target of rapamycin; HOTAIR, HOX transcript antisense RNA; OEA, ovarian endometrioid adenocarcinoma; RA, retinoic acid; HNSCC, head and neck squamous cell carcinoma; MAPK, mitogen-activated protein kinase; Igf1, insulin-like growth factor 1; AP-1, activator protein-1; TGCT, testicular germ cell tumor; hTERT, telomerase reverse transcriptase; EMT, epithelial-mesenchymal transition; TGF-β, Transforming growth factor β; cSCC, cutaneous squamous cell carcinoma; HIF-1α, hypoxia inducible factor-1.
